# Supplementary material for: Evidence supported by Mendelian randomization: impact on inflammatory factors in knee osteoarthritis
Source: Front Med (Lausanne). 2024 May 28;11:1382836. doi: 10.3389/fmed.2024.1382836 (PMC11165061; doi:10.3389/fmed.2024.1382836)
Supplement: Supplementary file 1 [file Data_Sheet_1.ZIP › data/GCST90274827_5e6/827.forest.pdf]

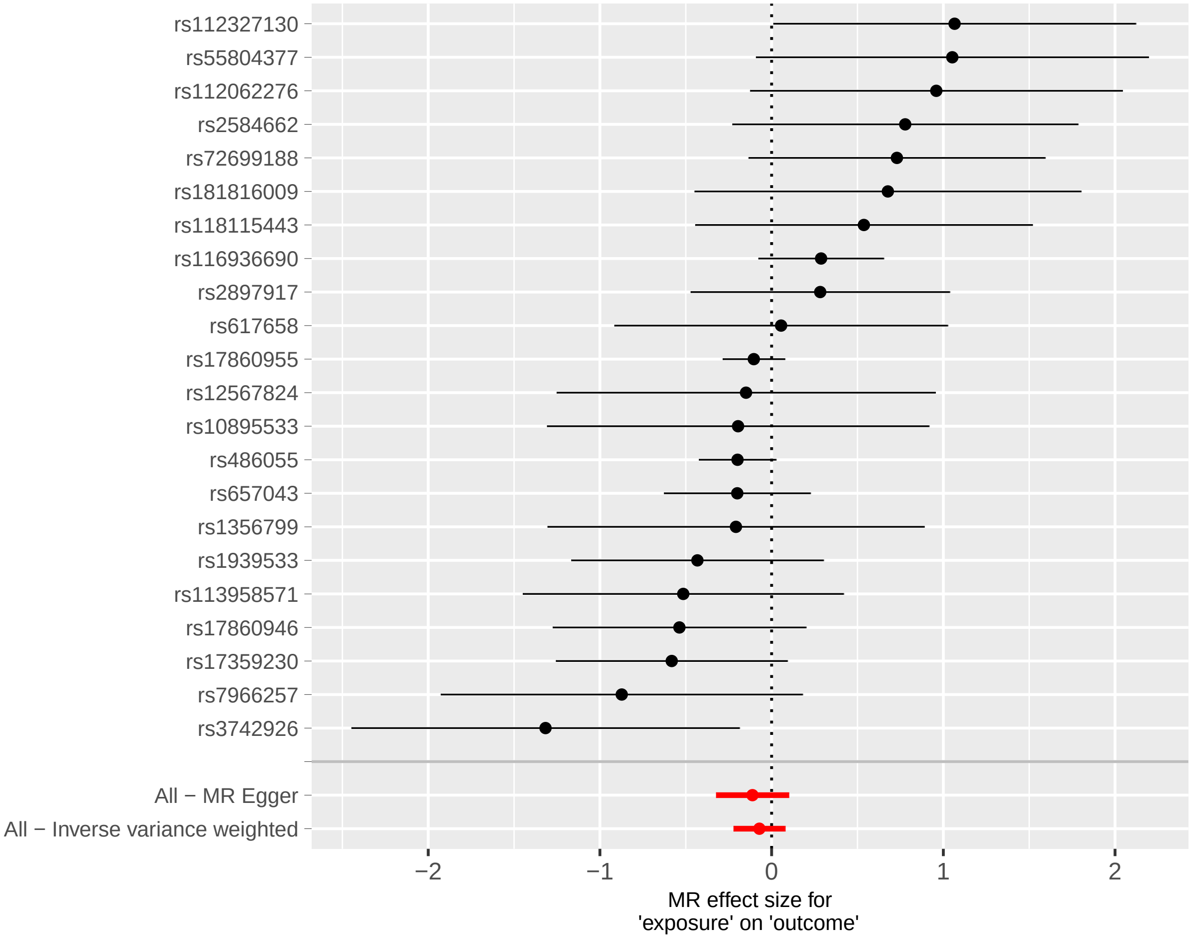

rs112327130
rs55804377
rs112062276
rs2584662
rs72699188
rs181816009
rs118115443
rs116936690
rs2897917
rs617658
rs17860955
rs12567824
rs10895533
rs486055
rs657043
rs1356799
rs1939533
rs113958571
rs17860946
rs17359230
rs7966257
rs3742926
All − MR Egger
All − Inverse variance weighted
−2
−1
0
1
2
MR effect size for
'exposure' on 'outcome'
